# Ethereal Vapor

**Published:** 1847-06

**Authors:** 


The following we have received from Dr. Brewster, of Portsmouth, N. H.

*Ethereal Vapor.*—There has been a current report, which is now established by a doubtless authority, that within a few weeks a young married lady, of delicate circumstances, called on a travelling dentist, in a town situated a few miles distant from Portsmouth, N. H., for the purpose of having a diseased tooth extracted. The dentist, regardless of her situation, and too ignorant to suspect the consequences awaiting his practice in the case, proposed the inhalation of *ether vapor,* as a preventive of the pain which would otherwise be consequent. She submitted, and the tooth was extracted. The effects of the gas on her muscular system produced so entire a disability of retention, that, in course of a few hours, and before she could leave the dentist's boarding place, *an abortive birth was the result.*

